# Influence of Psychological Separation on Master Graduates’ Career Maturity: Mediating Role of Occupational Self-Efficacy

**DOI:** 10.3389/fpsyg.2022.833577

**Published:** 2022-03-17

**Authors:** Ni Jianchao, Wang Yumei, Li Dongchen

**Affiliations:** ^1^School of Aerospace Engineering, Xiamen University, Xiamen, China; ^2^Institute of Education, Xiamen University, Xiamen, China; ^3^School of Sociology and Anthropology, Xiamen University, Xiamen, China

**Keywords:** career maturity, psychological separation, occupational self-efficacy, master graduates, mediating effect

## Abstract

This research adopts the career maturity as an indicator of one’s intention and ability in career development, and explores whether and how psychological separation from parents and occupational self-efficacy influence one’s career maturity. A structural equation model is constructed and tested with data from a survey of 584 master graduates in top universities across China. According to the study, it is founded that: (1) The psychological separation and occupational self-efficacy of master graduates are significantly and positively correlated with occupational self-efficacy, and career maturity, respectively. (2) Occupational self-efficacy plays a part of the mediating role between psychological separation and career maturity. (3) Master graduates with a high degree of psychological separation are more confident in achieving their career goals, more involved in the career selection process and have a higher level of career development. These findings will arouse the attention of the society to the master graduates’ career maturity to some extent.

## Introduction

The jobs that employers provide can no longer keep up with the speed and scale of graduate enrollment expansion, meanwhile, the group of highly educated master graduates has gradually lost its previous competitive advantage in the job market. Compared with undergraduates, master graduates experience heavy scientific research pressure. However, their employment expectations are overly high due to the lack of career preparation. Compared with doctoral students, master graduates face both academic and employment pressures, and their scientific research ability is slightly insufficient. Therefore, this research selects master graduates as the research object to explore the relationship between psychological separation, occupational self-efficacy, and career maturity, and hopes to provide targeted and operable theoretical basis for graduate career education, consultation and practice.

Employment is the foundation of personal development. Career development is an important task for college students in determining their career paths, meanwhile, career education is intended to convey career information and assist in their career planning (Park, 2015). Facing the mounting job competition pressure, an in-depth research is required for the university graduates’ job-hunting intention and behavior. Career maturity is an important indicator to measure the level of individual career development, referring to the degree to which career development tasks are completed (Ju and Shin, 2020).

Career maturity is an essential factor of career preparation in modern society, therefore, it is one of the main areas of career development guidance (Peng and Johanson, 2006). Relevant research shows that career maturity has a better performance in individual career development and behavior prediction (Crites, 1961), and individuals with a high level of career maturity are ready for the choice of profession (Karahan et al., 2021). Therefore, career maturity and its influencing factors have attracted much attention recently.

Furthermore, studies have also shown that family background factors (i.e., family economic status, parental expectations, family relationships, the intimacy between parents and children, etc.) and individual psychological factors (i.e., self-concept, locus of control, emotional intelligence, general self-efficacy, career decision-making self-efficacy, etc.) have certain impacts on career maturity (Blustein et al., 1991). The family background is an important factor for individual career development, and the healthy development of an individual’s career significantly depends on the degree of psychological separation between the individual and the native family (Lee and Hughey, 2001). The individual is the executor of career development and the individual’s psychological factors are closely related to career maturity. To clarify whether family factors will affect the career maturity of master graduates through individual factors, this research introduces the variable “psychological separation” which is closely related to the family system and the variable “occupational self-efficacy” of individual occupational psychology to explore the relationship between psychological separation, occupational self-efficacy and the career maturity of master graduates. In former literatures, there is little research on the relationship among the three variables of psychological separation, occupational self-efficacy and career maturity. In this research, several questions are raised: (1) How do psychological separation and occupational self-efficacy affect career maturity? (2) Does psychological separation affect career maturity by affecting individual occupational self-efficacy? If so, does the psychological separation affect career maturity entirely by influencing occupational self-efficacy, or does it affect career maturity partially by affecting the individual’s occupational self-efficacy? Focusing on above questions, the questionnaire survey method combining with the structural equation model and analysis methods such as bias-corrected self-sampling method is adopted in this research to clarify the relationship between psychological separation, occupational self-efficacy and career maturity. Moreover, most researches of career maturity are focused on undergraduates and vocational students, less attention is paid to graduate students, especially to master graduates. However, with the increasing number of master graduates, the graduate education has gradually moved from elite to popularization, corresponding researches become necessary to guild these students.

## Literature Review and Theoretical Hypothesis

Family environment is an important factor for individual personality formation and socialization. Marcia believes that the formation of an ego ideal occurs during adolescence, failure to complete this task leaves one at the mercy of an un-reconstructed superego formed in childhood, when the internalized parental figures are formidable characters in the child’s life, and they will seek out authorities upon whom they can depend for guidance in their adult lives (Kroger and Marcia, 2011). When an individual experiences the period of puberty, a series of psychological changes will occur along with physical maturity, resulting in a “sense of adulthood.” The individual’s sense of independence is gradually strengthened, and they hope to deal with their own affairs independently. Compared with childhood, their emotional dependence on their parents is greatly reduced, and their value judgment of right and wrong is gradually formed. In the graduate stage, individuals go out to study alone, which is an important period for individuals to obtain autonomy independent of their original family. Bloom believes that the success of psychological separation from parents will affect the healthy level of personality and social relations in adulthood (Stevens, 1981). As an important indicator to measure the quality of interaction between individuals and their families, psychological separation can reflect the degree of the individual separation from their parents’ dependence, gaining independence and autonomy, and forming individualization (Blos, 1979). In this study, psychological separation includes three aspects: emotional separation, attitude separation and behavior separation between individuals and their parents. Among them, emotional separation refers to the individual’s emotional independence. Individuals with a high level of emotional separation can emotionally get rid of their strong dependence on their parents and obtain autonomy. Behavior separation reflects the individual’s independence in behavior decision-making. Individuals with high level of behavior separation can make decisions and take actions independently without the help of their parents when dealing with their own affairs. Attitude separation shows the independence of individuals in the value system. Individuals with a high level of attitude separation have their own independent judgment in terms of morality. Moreover, individuals with higher psychological separation has higher autonomy while maintaining a harmonious emotional connection with their families, feeling active, can control their own lives and believe that they are excellent enough to achieve behavioral goals (Reid and Anderson, 1992). Research has found that the higher the degree of separation from the original family, the higher their sense of competence (Frank et al., 1988).

In 1977, Bandura, an American psychologist, first proposed the concept of self-efficacy. He believes that self-efficacy refers to the degree of individual confidence in whether they can use their ability to complete the target behavior (Bandura and Bandura, 1986). According to Bandura’s definition of self-efficacy, self-efficacy is for a specific task. Therefore, once the concept of self-efficacy was put forward, it has attracted the attention of various fields. In 1981, Betz and Hackett first applied the theory of self-efficacy to the occupational field and put forward the concept of occupational self-efficacy (Betz and Hackett, 2011). According to Bandura’s definition of self-efficacy, occupational self-efficacy can be understood as an individual’s confidence and belief in his ability to complete a specific occupational behavior. Occupational self-efficacy runs through a person’s career, from career preparation to career choice, career adaptation to the end of career, and plays a positive role in individual career development. As the career decision-making self-efficacy can effectively promote individuals to improve their career maturity level (Taylor and Betz, 1983). According to the concept of self-efficacy, career decision-making self-efficacy can be understood as the degree of individual confidence in their own ability in the process of career decision-making. On this basis, this research believes that occupational self-efficacy may be an intermediary variable of psychological separation affecting career maturity.

### Psychological Separation and Occupational Self-Efficacy

There is little research on the relationship between psychological separation and self-efficacy among graduate students, especially occupational self-efficacy. Some scholars have explored the relationship between psychological separation and self-efficacy on high school students. For example, Korean scholar Kang (2016) took high school students of physical education as the research object, and found that psychological separation has a positive effect on career decision-making self-efficacy. YoungKeun et al. (2011) studied the relationship among psychological separation, gender identity, academic performance, and occupational self-efficacy of Korean high school students, it is found that psychological separation can positively predict the occupational self-efficacy. The higher the level of psychological separation between high school students and their parents, the higher the level of occupational self-efficacy (Kim et al., 2011).

Erikson (1968) believes that physical and psychological separation is one of the important development tasks of individuals: to establish a clear self-concept, the individual needs to get rid of the internal dependence on their parents and become psychologically independent. Individuals with a high level of psychological separation while maintaining good emotional connections with others are able to be independent, having more sense of self-identity, higher self-esteem and greater confidence in themselves in accomplishing specific goals. A successful psychological separation process can also help the individual to establish independence and autonomy from their parents, making it easier to accumulate successful experiences, build a positive psychological state, and obtain a strong sense of self-efficacy. Accordingly, this research puts forward the following hypothesis:

**Hypothesis h1:** Psychological separation may affect occupational self-efficacy.

### Occupational Self-Efficacy and Career Maturity

Occupational self-efficacy refers not only to the self-efficacy on the professional content, but also to the self-efficacy on career behavior process, that is, the individual’s belief in completing career behaviors and achieving behavior goals, such as career exploration, career problem solving and career decision-making, etc.

It is pointed out in Bandura’s research that people with higher occupational self-efficacy will be more confidence in their careers, can carry out more active job-hunting behaviors and become much easier to make career decisions to achieve successful employment (Bandura et al., 1997).

Occupational self-efficacy exhibits and non-negligible impact on various aspects of individual career behavior, for example, career decision-making self-efficacy is significant negatively correlated with career uncertainty (Reid and Anderson, 1992), and is positively correlated with career exploration behavior (Turner and Lapan, 2005), high-level career decision-making self-efficacy is related to diversified career identity and broader career exploration activities (Gushue, 2006). Domestic researches on the relationship between occupational self-efficacy and career maturity are paid more attention to the impact of occupational self-efficacy and career decision-making self-efficacy on career maturity. There is apparent internal relationship among occupational self-efficacy, career decision-making self-efficacy and career maturity. Studies have shown that career decision-making self-efficacy plays a partial intermediary role between proactive personality and career maturity (Ye et al., 2020).

In other words, individuals with high occupational self-efficacy, high self-evaluation, clear career goals and excellent problem-solving skills will always accompany with an impact on career maturity. That is, the higher the occupational self-efficacy is, the more objective of the individual’s self-evaluation, the more confident, active, independent, and flexible they will be in career decision-making. As a result, more career knowledge and career world knowledge will be accumulated, that is, the higher their career maturity will be. Accordingly, this research puts forward the following hypothesis:

**Hypothesis h2:** Occupational self-efficacy may affect career maturity.

### Psychological Separation and Career Maturity

Unsolved psychological tasks between the adolescents and their families will bring in extremely harmful affect to the individual’s mental health and personality development, and finally affect the subsequent career development.

Erikson believes that human psychological development is a lifelong process, and numerous different development tasks are required to be solved during each stage. Furthermore, residuary questions of the previous stage will significantly affect the next development stage (Betz and Hackett, 2011).

Larson (1995) pointed out that factors in a family system, such as excessive participation of parents and rigid family rules, have more influence on individual career decision-making than the influence of socioeconomic status and education. In addition, studies have also found that the independence degree between children and their parents will also affect their career investment, which in turn affects their career development level (Lucas, 1997).

Psychological separation has an important impact on career maturity. Generally speaking, a higher degree of psychological separation, strong independence and autonomy can encourage the initiative to accumulate career knowledge, collect more job-hunting information, improve their job-hunting ability, perfect their career planning, and obtain a higher career maturity (Powell and Luzzo, 2011). Accordingly, this research puts forward the following hypothesis:

**Hypothesis h3:** Psychological separation may affect career maturity.

### The Mediating Effect of Occupational Self-Efficacy

As mentioned above, individuals with better psychological separation are more independent and autonomous and have a stronger sense of self-identity when facing career choices. They understand themselves more clearly, have a specific positioning of career goals, can make appropriate attribution in the face of success and failure and are more confident in achieving career goals. Therefore, they will actively explore the career world, collect career information and master relevant career knowledge. They have stronger resistance to frustration, more excellent ability to solve problems and better career maturity. Based on these features, this research believes that occupational self-efficacy may be a potential mediating variable between psychological separation and career maturity, correspondingly, following hypothesis is made:

**Hypothesis h4:** Occupational self-efficacy plays a mediating role between psychological separation and career maturity.

**FIGURE 1:**
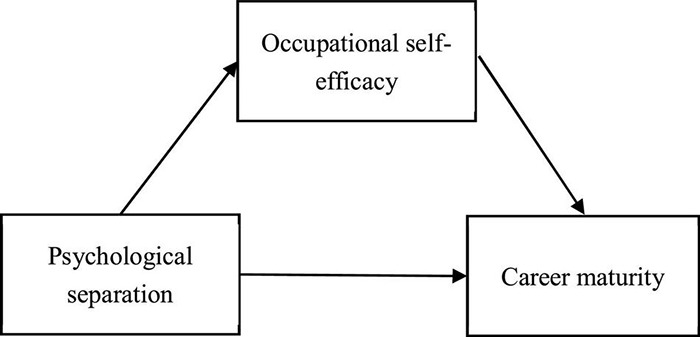
Hypothetical model of the relationship between psychological separation and career maturity with occupational self-efficacy as an intermediary variable.

To sum up, master graduates is the target population of this research to explore the relationship between master graduates’ psychological separation, occupational self-efficacy and career maturity. The relationship model among the three factors is shown in Figure 1.

## Materials and Methods

### Participants

A total of 600 master graduates from Xiamen University, Beijing University of Aeronautics and Astronautics, Lanzhou University, Chongqing University, Guangxi University, Fuzhou University and some top universities across China (The universities are geographically distributed, involving different levels) were selected as the participants. 584 valid questionnaires were finally obtained. The basic characteristics of the master graduates are shown in Table 1. Obviously, the students are evenly distributed in demographic variables in gender, major, and place of birth, therefore, the sample is representative.

**TABLE 1:** Distribution of the respondents by demographic characteristics (*N* = 584).

Variables		Count	Percentage
Gender	Male	339	58.0
	Female	245	42.0
Major	Science	114	19.5
	Engineering	310	53.1
	Liberal arts	140	24.0
	Others disciplines	20	3.4
Only child	Yes	250	42.8
	No	334	57.2
Residence	Urban	281	48.1
	Rural	303	51.9
	Total	584	100.0

### Procedure

#### Career Maturity Scale

Self-compiled “Career Maturity Questionnaire for Master Graduates” is designed to measure the career maturity of master graduates. The questionnaire is divided into three sub-questions, including career choice ability questionnaire, career choice attitude questionnaire and career choice knowledge questionnaire, a total of 60 questions. Especially, the career choice ability questionnaire is composed of five factors: career self-cognition, career exploration, career goal positioning, career decision-making, and career problem solving. The career choice attitude includes the other five factors: Career confidence, career independence, career initiative, career flexibility and career frustration resistance. Then, the career choice knowledge is composed of two factors: Career knowledge and career world knowledge. Career choice ability includes 26 items such as “I can timely capture the latest information of the career I want to engage in,” “when I encounter difficulties in finding a job, I will find ways to actively solve them” etc. Career choice attitude includes 23 items such as “I am full of hope for my future career development,” “I will actively learn from the experience of successful people to plan my career life” etc. Career choice knowledge includes 11 items such as “my professional skills are not proficient,” “I know the years of education required for different occupations” etc. Participants are classified by a 4 –point Likert scale (1 = very inconsistent, 4 = very consistent). Among the questionnaire, the items of 2, 7, 8, 12, 20, 21, 25, and 33 are entitled reverse directions. After recalculating the reverse, the reliability of three dimensions are measured. The Cronbach’s α of the three dimensions are between 0.77 and 0.91, indicating that the self-compiled scale has a good consistency in measuring these three dimensions. Then the question scores in different dimensions of the questionnaire are summed and averaged to obtain the scores of the three dimensions. The higher the score, the higher the maturity of the sample object in that dimension.

#### Psychological Separation Scale

The psychological separation scale is selected from the questionnaire compiled by Wu (2012), which is divided into father sub-questionnaire and mother sub-questionnaire, a total of 41 questions. Both sub-questionnaires include three dimensions: emotional separation, behavior separation and attitude separation. Father’s emotional separation includes 8 items such as “if I am separated from my father for too long, I will miss him very much” etc. Father’s behavior separation includes 6 items such as “I will seek my father’s support when making decisions or making plans” etc. Father’s attitude separation includes 6 items such as “I often have inconsistent views with my father in interpersonal communication” etc. Mother’s emotional separation includes 10 items such as “I hope I can get more encouragement and support from my mother” etc. Mother’s behavior separation includes 6 items such as such as “I often decide whether to do something according to my mother’s consent” etc. Mother’s attitude separation includes and 5 items such as “my mother and I usually agree on current social problems” etc. Participants rate the items on a 4 –point Likert scale. Among the questionnaire, the items of 2, 5, 7, 12, 18, 20, 22, 24, 25, 26, 31, 38, and 41 are entitled reverse directions. After recalculating the reverse, the reliability of three dimensions are measured. The Cronbach’s α is greater than 0.8, an acceptable consistency is obtained. Therefore, the corresponding question scores of different dimensions in the questionnaire are summed up, and the mean value is taken to obtain the scores of six dimensions. The higher the score, the higher the degree of psychological separation of the sample object in this dimension.

#### Occupational Self-Efficacy Scale

The occupational self-efficacy scale used in this paper is from the research of Schyns and Collani (2002), a total of nine questions, which is used to measure the degree of confidence of master graduates in completing corresponding professional behaviors and achieving professional goals. The questionnaire is one single dimension, including “when I make plans concerning my occupational future, I can make them work,” “I can always manage to solve difficult problems in my job if I try hard enough” etc. In order to be consistent with other scales, Participants are rated the items on a 4–point Likert scale. (1 = completely disagree, 4 = very agree). The Cronbach’s α is 0.8109. All questions are summed up and the mean value is taken. The higher the score, the stronger the occupational self-efficacy.

### Data Analysis

Firstly, the Harman single factor test method was used to test the common method deviation of the data. It is found that the total variance explained by the first common factor is 22.745%, which is less than the critical value of 40%. Therefore, the data in this research does not have the common method deviation problem. After then, SPSS23.0 and AMOS24.0 were used to carry out correlation analysis, reliability analysis and confirmatory factor analysis (CFA) to test the reliability of each questionnaire and the discriminative validity of each variable. Finally, the structural equation model and bias-corrected bootstrap are used to verify the research hypothesis.

## Research Results

### Confirmatory Factor Analysis and Correlation Analysis

The CFA was carried out using AMOS24.0 to test the validity of each variable in this research. The results are shown in Table 2. As can be seen from Table 2, the combined reliability (CR) of each variable is in the range of 0.681–0.928, which is basically close to or greater than 0.65. The average variance extraction (AVE) is also more than 0.5. Therefore, it can be considered the above variables have good convergence validity to measure the target latent variables.

**TABLE 2:** Convergence validity test of model variables.

Latent variable	Observed variable	Std.	Unstd.	S.E.	*t*-value	*P*	SMC	CR	AVE
Psychological separation	Separation of mother attitude	0.862	1				0.743	0.681	0.815
	Separation of mother behavior	–0.201	–0.236	0.049	–4.847	***	0.04		
	Separation of mother emotion	0.715	0.611	0.037	16.512	***	0.511		
	Separation of father attitude	–0.246	–0.218	0.039	–5.569	***	0.061		
	Separation of father behavior	0.683	0.841	0.053	15.823	***	0.466		
	Separation of father emotion	0.882	0.723	0.044	16.551	***	0.778		
Occupational self-efficacy	Q1	0.219	1				0.048	0.887	0.596
	Q2	0.657	1.082	0.218	4.972	***	0.432		
	Q3	0.639	1.303	0.263	4.946	***	0.408		
	Q4	0.727	1.371	0.273	5.021	***	0.529		
	Q5	0.684	1.211	0.243	4.992	***	0.468		
	Q6	0.682	1.094	0.219	5.003	***	0.465		
	Q7	0.746	1.206	0.239	5.051	***	0.557		
	Q8	0.728	1.233	0.246	5.021	***	0.53		
	Q9	0.746	1.119	0.223	5.025	***	0.557		
Career maturity	Career choice ability	0.946	1				0.895	0.928	0.927
	Career choice attitude	0.888	0.799	0.022	35.918	***	0.789		
	Career choice knowledge	0.867	0.957	0.029	33.067	***	0.752		

***means at the significant level of 0.001.

In addition, Pearson correlation analysis was implemented on the main variables, and the square root of the variable’s AVE and the correlation coefficient of the variable and any other variable were compared to test the discriminative validity. Obviously in Table 3, the variables in this research basically have a favorable discriminative validity.

**TABLE 3:** Pearson correlation and AVE square root value.

	Psychological separation	Occupational self-efficacy	Career maturity
Psychological separation	0.627		
Occupational self-efficacy	0.246***	0.555	
Career maturity	0.296***	0.661***	0.900

The diagonal numbers are the square root value of AVE. ***means at the significant level of 0.001.

The correlation coefficients among the variables are shown in Table 3. Psychological separation is significantly positively correlated with occupational self-efficacy and career maturity, and occupational self-efficacy is significantly positively correlated with career maturity. The results meet the theoretical expectations.

### The Hypothesis Test

Structural equation model was used to test the effect of psychological separation on career maturity. In this model, the psychological separation is designed as the independent variable, the career maturity as the dependent variable and occupational self-efficacy as the intermediary variable. The fitting index of the model is tested. Since psychological separation is divided into father psychological separation and mother psychological separation, both of the two separations include three dimensions. Career maturity is also divided into three dimensions: career choice ability, career choice attitude, and career choice knowledge. Each dimension is packaged separately, meanwhile, occupational self-efficacy is not packaged, as a result, the model contains a total of three latent variables and 18 manifest variables. The results of the measurement model show that the factor loading of each index is significant (*P* < 0.001), indicating that each latent variable can be well represented by its own index.

**FIGURE 2:**
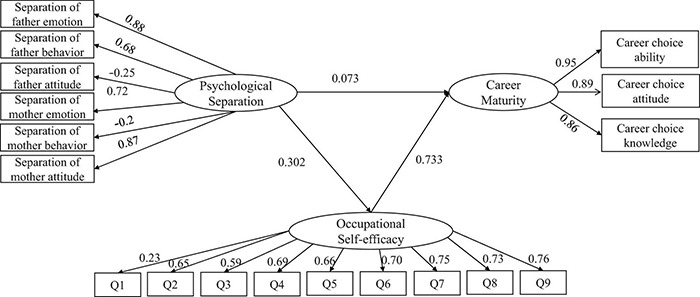
The mediating effect of occupational self-efficacy between psychological separation and career maturity.

According to the intermediary effect test procedure proposed by existing researchers (Wen et al., 2004), a structural equation model is constructed. The statistical results are shown in Figure 2, obviously, the model is well fitted (χ^2^/df = 2.787, RMSEA = 0.055, GFI = 0.934, NFI = 0.94, RFI = 0.922, IFI = 0.961, TLI = 0.949, CFI = 0.96), indicating that the hypothetical model and the collected data matches well with each other.

It can be seen from Table 4 that the non-standardized path coefficient of psychological separation on occupational self-efficacy is 0.295, and the *P*-values are all less than 0.01, which means that for each unit of psychological separation, the occupational self-efficacy will increase by 0.295 units, respectively, verifying h1 is true. The non-standardized path coefficient of occupational self-efficacy to career maturity is 0.578, and the significance *P*-value is less than 0.05, which means that the intensity of career maturity will increase by 0.578 units for each unit of psychological separation, therefore, h2 is true. The non-standardized path coefficient of psychological separation to career maturity is 0.056, and the significance *P*-value is less than 0.05, which means that the intensity of career maturity will increase by 0.056 units for each unit of psychological separation, h3 is true.

**TABLE 4:** Path coefficient.

Variables	Relationship direction	Variables	Non-standardized path coefficient	Standardized path coefficient	*S.E.*	*C.R.*	*P*	Hypothesis
Occupational self-efficacy	**< —**	Psychological separation	0.295	0.302	0.044	6.638	***	True
Career maturity	**< —**	Occupational self-efficacy	0.578	0.733	0.037	15.422	***	True
Career maturity	**< —**	Career maturity	0.056	0.073	0.025	2.266	0.023	True

***means at the significant level of 0.001.

The Bias-corrected Bootstrap method, which is widely accepted in academia is employed in this paper to test the mediating role of occupational self-efficacy between psychological separation and career maturity. The bootstrap is set to be 5,000 times in AMOS24.0 and the confidence interval is taken as 95% to test the mediation effect, the results are shown in Table 5. In the analysis of the mediating effect of psychological separation on career maturity, the indirect effect β is 0.171 (BootLLCI = 0.103 BootULCI = 0.239), the confidence interval of the indirect effect does not contain 0, indicating that there is an intermediary role of occupational self-efficacy between psychological separation and career maturity; the confidence interval of the direct effect does not contain 0, indicating that the mediating effect of occupational self-efficacy between psychological separation and career maturity is partial, and the amount of mediating effect (indirect effect/total effect) is (0.171/0.227 = 75.3%), h4 is true.

**TABLE 5:** Mediation effect test.

Effect type	Estimated value	95% Confidence interval	
		Lower limit	Upper limit
Indirect effect	0.171	0.103	0.239
Direct effect	0.056	0.005	0.117
Total effect	0.227	0.142	0.313

## Discussion

This research focuses on examining the mediating role of occupational self-efficacy, and the relationship between master graduates’ psychological separation and career maturity. Data analysis shows that there is a significant positive correlation between the psychological separation of master graduates and career maturity. Psychological separation cannot only directly affect the career maturity of master graduates, but also indirectly affect career maturity level by affecting occupational self-efficacy.

### The Relationship Between Psychological Separation and Career Maturity

Correlation analysis results show a significant positive correlation between psychological separation and career maturity: a higher level of psychological separation will always lead to a higher career maturity. Structural equation model analysis results also show that psychological separation can significantly promote career maturity. This is consistent with the previous research results. O’Brien (1996) believe that psychological separation has a significant impact on career maturity. The degree of separation between children and family has a significant impact on youth’s career decision-making, and a better level of psychological separation will reduce youth’s career decision-making difficulties (Lopez and Andrews, 2014). Generally speaking, individuals with high psychological separation have higher career maturity.

### The Relationship Between Psychological Separation and Occupational Self-Efficacy

According to Erikson’s eight-stage theory of personality development, self-identity is determined around 18 years old, and some researchers believe that individual identity will become more and more mature with age (Marcia, 1966). Master graduates are generally around 25 years old and face important development tasks such as career choices. Their psychological separation from their parents will affect their self-identity maturity, which in turn affects their occupational self-efficacy development. Correlation analysis results show that there is a significant positive correlation between psychological separation and occupational self-efficacy, and the structural equation model analysis results also show that psychological separation can significantly promote the occupational self-efficacy of master graduates. In the past, few scholars have conducted research on the relationship between the two variables. Psychological separation is the main content of adolescent parent-child relationship. It is a process during which individuals seek independence, autonomy and self-perception on the basis of forming an intimate emotional connection with their parents. Erikson mentioned in his research that in the process of individualization, individuals need to keep a distance from their parents and avoid being controlled by their parents in order to develop their personality. When the individual successfully develops individualization which is suitable for his age, he will gain autonomy which is conducive to the development of individual self-concept and sense of identity (Betz and Hackett, 2011). Individuals who can separate from the family perceive that they can freely control themselves and the environment, smoothly resolve the identity crisis and form self-identity (Mjn et al., 1999), accompanying with a higher sense of self-identity, a positive self-concept, be able to have a clearer sense of self-identity and feel more confident in the ability to accomplish goals through their own behavior, that is, have a higher sense of occupational self-efficacy.

### The Relationship Between Occupational Self-Efficacy and Career Maturity

Correlation analysis results show that the occupational self-efficacy of master graduates is significantly positively correlated with their career maturity. A higher occupational self-efficacy degree will lead to a higher career maturity. Structural equation model analysis results also show that occupational self-efficacy has a positive effect on career maturity, which is basically consistent with previous studies (Erikson, 1968). Master graduates with higher occupational self-efficacy have a higher degree of career maturity.

### The Mediating Role of Occupational Self-Efficacy Between Psychological Separation and Career Maturity

Structural equation model and bias-corrected self-sampling method analysis results show that psychological separation not only directly affects career maturity, but also indirectly affects career maturity by affecting occupational self-efficacy. Master graduates with a high levels of psychological separation have a value system independent to their parents on moral, ideological and social issues, can maintain the independence of emotional and behavioral decision-making, have stronger self-confidence in achieving career goals, and can objectively conduct self-analysis and evaluation. They can clarify their career goals, learn more about career exploration, master career and job related knowledge. In addition, they can update information in time, understand and discover the needs and variables of the external environment and make appropriate adjustments in time. They can adhere to their career goals, which will help to improve their career maturity and development level even if they encounter difficulties.

On the contrary, master graduates with low levels of psychological separation rely too much on their parents and help of their parents when it comes to their own affairs, have low independence in value system and behavioral decision-making, and rely too much on the. They are not confident enough to achieve their career goals, easy to deny themselves, unable to objectively and comprehensively evaluate themselves and could hardly choose a suitable career. They cannot proactively explore and learn relevant knowledge. When faced with difficulties, their attitude is passive and unable to make timely adjustments according to the actual environment, and they are easy to relax and give up. These behaviors hinder the development of their career maturity.

### Limitations and Future Directions

There are still limitations on current study. Firstly, the sampling source has certain limitations, and the sample size is relatively small, contemplation of geographic area and courses is relatively insufficient, so it is difficult to generalize the results. Secondly, with convenience sampling, there may be selection bias and potential threats. Finally, due to time constraints, questionnaire survey was conducted without intervention, and the use of Likert questions forced respondents to choose answers instead of open response. Therefore, as part of future research, follow-up research should be designed and implemented using multiple data collection methods. Longitudinal research should be carried out after conducting questionnaire survey. Future research should include longitudinal data or experimental methods to verify their relationships, and extend to other types of master’s degrees and other geographical realities, and analyze in a factorial way to better cover the universe studied.

## Conclusion

This research explored the relationship between the master graduates’ psychological separation and their career maturity while verifying the mediating role of occupational self-efficacy. The results show that: (1) Psychological separation has a significant effect on the occupational self-efficacy and career maturity of master graduates, the more they believe that they can achieve their career goals, the higher their level of career maturity will be. (2) Occupational self-efficacy also has a significant effect on the career maturity of master graduates. The more they believe that they can achieve their career goals, the higher their career maturity level will be. (3) Occupational self-efficacy plays a partial intermediary role between the psychological separation and occupational maturity of master graduates. Psychological separation cannot only directly affect the career maturity of master graduates, but also indirectly affect the career maturity through occupational self-efficacy. Specifically, master graduates with a high level of psychological separation are more independent and autonomous, and they have stronger beliefs in achieving certain behavioral goals, therefore, they will invest in career behavior more actively, so as to improve their career maturity level, and win more and better employment opportunities for themselves.

### Research Value

The theoretical contributions of this research as follows: Firstly, enrich the research on the relationship between psychological separation and occupational self-efficacy. Occupational self-efficacy plays an important role in individual career development, but there is little domestic research on the impact of psychological separation. This research explored the influence of psychological separation on occupational self-efficacy, enriched the related research of psychological separation in the field of career development and further promoted the localized research of psychological separation occupational self-efficacy. Secondly, it analyzes the relationship between psychological separation and career maturity. This research uses occupational self-efficacy to explain the mechanism of psychological separation on career maturity, emphasize the mediating effect of occupational self-efficacy, clarify the logical relationship between psychological separation and career maturity, and further enrich the research on career maturity.

This research will arouse the attention of the society to the career maturity of master graduates to some extent. The research results on the influence mechanism of psychological separation on career maturity remind us that the role of occupational self-efficacy between psychological separation and career maturity cannot be ignored in graduate career development education. Colleges and universities should follow the development law of occupational self-efficacy, formulate scientific educational measures, cultivate a good sense of occupational self-efficacy of master graduates, and improve the career development level of master graduates. In addition, in clinical practice, therapists should focus on helping master graduates realize the independence of emotion, attitude and behavior on the premise of maintaining emotional connection with their parents, which cannot only directly improve the career maturity of master graduates, but also indirectly affect the career maturity by affecting the level of occupational self-efficacy of master graduates.

## Data Availability Statement

The raw data supporting the conclusions of this article will be made available by the authors, without undue reservation.

## Author Contributions

NJ and WY designed the study and wrote the manuscript. WY and LD analyzed the data. NJ and WY modified the manuscript. All authors have read and agreed to the published version of the manuscript.

## Conflict of Interest

The authors declare that the research was conducted in the absence of any commercial or financial relationships that could be construed as a potential conflict of interest.

## Publisher’s Note

All claims expressed in this article are solely those of the authors and do not necessarily represent those of their affiliated organizations, or those of the publisher, the editors and the reviewers. Any product that may be evaluated in this article, or claim that may be made by its manufacturer, is not guaranteed or endorsed by the publisher.
